# Selection and Expression Profiles of Reference Genes in Mouse Preimplantation Embryos of Different Ploidies at Various Developmental Stages

**DOI:** 10.1371/journal.pone.0098956

**Published:** 2014-06-13

**Authors:** Yanli Gu, Xinghui Shen, Dongjie Zhou, Zhendong Wang, Na Zhang, Zhiyan Shan, Lianhong Jin, Lei Lei

**Affiliations:** Department of Histology and Embryology, Harbin Medical University, Harbin, Heilongjiang Province, China; State Key Laboratory of Reproductive Biology, Institute of Zoology, Chinese Academy of Sciences, China

## Abstract

Real-time reverse transcription quantitative polymerase chain reaction (qPCR) has become the most frequently used system for studies of gene expression. Manystudies have provided reliable evidence that the transcription levels of reference genes are not constant at different developmental stages and in different experimental conditions. However, suitable reference genes which are stably expressed in polyploid preimplantation embryos of different developmental stages have not yet been identified. Therefore, it is critical to verify candidate reference genes to analyze gene expression accurately in both diploid and polyploid embryos. We examined the expression levels of 12 candidate reference genes in preimplantation embryos of four different ploidies at six developmental stages. Stability analysis of the reference genes was performed by four independent software programs, and the stability of three genes was evaluated by comparison with the *Oct4* expression level during preimplantation development in diploid embryos. The expression levels of most genes in the polyploid embryos were higher than that in the diploid embryos, but the increasing degree were disproportionate with the ploidies. There were no significant difference in reference gene expressions among embryos of different ploidies when they reached the morula stage, and the expression level remained flat until the blastocyst stage. *Ubc*, *Ppia*, and *Pgk1* were the three most stable reference genes in diploid and polyploid embryos.

## Introduction

Gene expression studies in tissue or cell samples are dependent on the use of appropriate reference genes. Some researchers have proposed assuming that reference genes which are expressed at a constant level in tissues or at all stages of development are unaffected by experimental treatments. To date, no reference gene is universally applicable in gene expression studies for all tissues or cell types [1]–[3]. Most standardizations use commonly known reference genes, such as *β-actin*, *Gapdh*, and *18s* [1], [4]. However, a great many studies have provided powerful demonstration that the transcription levels of reference genes are not constant at different developmental stages and in different experimental conditions [4]–[6]. Thus, normalization of data using these reference genes could result in false conclusions. Therefore, it is critical to verify candidate reference genes to analyze gene expression accurately. To study gene expression in preimplantation embryos, the reference genes should be expressed stably at various developmental stages, and the variation in expression levels should be small, but not immutable. These levels should gradually increase as embryonic development progresses and with the increasing numbers of cells.

Preimplantation embryo is important model for evaluation of oocyte quality and disease study. So, the expression of genes in preimplantation embryo was usually to be checked. However, it is become difficult to assay the gene profiling in preimplantation embryo because the amount of mRNA in preimplantation embryos is variable for most genes, including reference genes [7]. However, a prerequisite for the usefulness of normalization is that the expression level of the reference genes does not vary markedly through preimplantation development or in response to different experimental conditions. The cells comprising the embryo are unlike cell lines and single-organ tissues, they have inherently a wildly heterogeneous nature, which induces more significant variation in endogenous biological processes and in the sensitivity of embryonic samples [8]. Therefore, normalization is required to avoid intra- and interassay variations. A number of studies addressed this issue by evaluating the reference genes of preimplantation embryos of different species, including rabbit [9], equine [10], and bovine [11], [12] embryos. In mouse studies, only few evaluations of stability analysis of the reference genes were carried out in diploid embryos [13], [14].

However, suitable reference genes which are stably expressed in various ploidies of preimplantation embryos have not yet been identified. Polyploid formation is an abnormal chromosomal phenomenon that has a low natural incidence. Fortunately, polyploid embryos can be produced in the laboratory for research. Mouse tetraploid (4N), hexaploid (6N), octoploid (8N), and even hexadecaploid (16N) embryos can be produced by electrofusion of blastomeres at the 2C stage [15], [16]. Many studies have focused on tetraploids, which are commonly used to rescue embryonic lethality as a result of defective extraembryonic phenotypes in laboratory mouse strains. There have been few studies that have measured and compared gene expression in tetraploids with diploids in mammalian cells. Studies have shown that 4N whole-genome expression levels and malate dehydrogenase (MDH) activity in 4N cells were not simply double that of 2N cells. Most gene expression levels were maintained at levels similar to the 2N, and the expression of only a few genes changed [17]–[20]. These results were derived from tetraploid mouse embryos at the morula and blastocyst stages. However, the reference gene expression patterns during development in earlier preimplantation in polyploid embryo stages are not yet clear.

We searched the recent literature and selected 12 candidate genes: *β-actin*, *Gapdh*, *H2afz*, *Tbp*, *Hprt*, *Ywhaz*, *Pgk1*, *Ubc*, *Ppia*, *Ppib*, *16s*, and *18s*. Most of these genes belong to different functional classes and should not be coregulated, thus providing a reliable method of normalizing qPCR expression data. We examined the expression levels of these 12 candidate reference genes in preimplantation embryos of four different ploidies (diploid, 2N; tetraploid, 4N; hexaploid, 6N; and octoploid, 8N) at six developmental stages (1-cell, 1C; 2-cell, 2C; 4-cell, 4C; morula, Mo; early blastocyst, EB; and late blastocyst, LB). Stability analysis of reference genes was performed using four independent software programs, namely geNorm, NormFinder, the comparative delta-Ct method, and RefFinder. Using these methods, we ranked the reference genes according to their stability and selected the most stable reference genes in diploid and polyploid embryos.

## Methods

### Animals

Animals in the experiments were 6–8-week-old ICR female and male mice (*Mus musculus*) purchased from Beijing Vital River Laboratory Animal Co. They were kept under conditions of 14 h light/10 h darkness with food and water. This study was carried out in strict accordance with the recommendations in the Guide for the Care and Use of Laboratory Animals. The protocol was approved by the Institutional Research Board of Harbin Medicine University (HMUIRB20130016). All surgery was performed under sodium pentobarbital anesthesia, and all efforts were made to minimize suffering.

### Preparation of mouse preimplantation embryos of different ploidies

#### Collection of diploid (2N) embryos

Each female mouse was superovulated by intraperitoneal injection of 5.0 IU pregnant mare serum gonadotropin (PMSG, NSH, China) and 7.5 IU human chorionic gonadotropin (hCG, NSH, China) given 48 h apart. Then the females were caged individually with males of the same strain. Mating was ascertained by the presence of a vaginal plug the next morning. The females were sacrificed 17 h after hCG injection. Zygotes were collected from the ampullae of the oviducts. Cumulus cells were removed from the zygotes with 300 µg/ml hyaluronidase (Sigma, H4272) in droplets of HEPES medium. Denuded zygotes were picked up and kept in new droplets of HEPES-buffered CZB medium (HEPES-CZB), covered with sterile mineral oil (Fisher, O121-20), and then cultured at 37°C in an atmosphere of 5% CO_2_ until use.

#### Preparation of tetraploid (4N) embryos

The superovulation and caging procedures were the same as those described above. The female mice were humanly killed at 42–46 h after hCG injection. Embryos at the 2C stage were flushed from the oviducts and incubated in potassium simplex optimized medium (KSOM) under paraffin oil at 37°C in an atmosphere of 5% CO_2_ in air until electrofusion. To generate tetraploid embryos, the recovered 2C embryos were arrayed in a fusion chamber filed with 275 mM mannitol supplemented with 0.1 mM MgSO_4_·7H_2_O, 0.05 mM CaCl_2_·2H_2_O, and 3 mg/ml bovine serum albumin (BSA). Fusion was induced by two 1.2 kV/cm (DC) pulses for 80 µs using a BTX2001. After electrofusion, the embryo was washed three times in HEPES-CZB, and incubated in KSOM for 30 minutes; fusion usually takes place within half an hour. Then the fused embryos were cultured at 37°C in an atmosphere of 5% CO_2_ in air.

#### Obtaining hexaploid (6N) embryos

According to the method described previously [15], the 2N-2C embryos and 4N-2C embryos were placed in HEPES medium containing 5 µg/ml cytochalasin B (CB). The procedure involves taking out one blastomere from a 2N-2C embryo and one blastomere from a 4N-2C embryo and swapping the two blastomeres, which results in two 2N/4N embryos. After swapping the blastomeres using this method, the 2N/4N embryo pairs were fused as mentioned above. Then the fused embryos were cultured in KSOM medium under paraffin oil at 37°C in an atmosphere of 5% CO_2_ in air.

#### Obtaining octoploid (8N) embryos

When the tetraploid embryos developed to the 2C stage, electrofusion was performed again to produce octoploid embryos, using the fusion conditions described above. The fused embryos were cultured in KSOM medium under paraffin oil at 37°C in an atmosphere of 5% CO_2_ in air.

**Figure 1:**
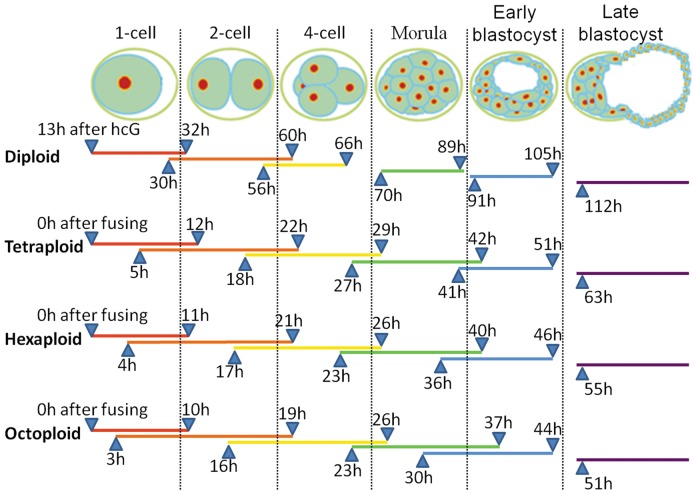
The developmental schedule of diploid, tetraploid, hexaploid, and octoploid preimplantation embryos. The developmental schedule of diploid embryos was counted from the hCG injection, and the developmental schedules of the tetraploid, hexaploid, and octoploid embryos were counted from 0.5-cell; orange, 2-cell; yellow, 4-cell; green, morula; blue, early blastocyst; purple, late blastocyst. The overlapping part represents the time period in which the embryos of two developmental stages coexist.

The developmental schedule for diploid preimplantation embryos is more consistent than the schedules for the polyploid embryos, so we first confirmed the schedule of embryonic development before collecting samples (Figure 1). We chose an intermediate time point in each developmental stage to ensure that more than 80% of the embryos were in the same developmental stage. Finally, the embryos of different ploidies and different developmental stages were washed three times in DEPC-PBS, collected individually in pools of 20 embryos, and stored at −80°C until mRNA extraction.

### mRNA isolation and cDNA reverse transcription

The messenger RNA (mRNA) was isolated from each group using Dynabeads mRNA Direct Kit (Invitrogen, Cat. No. 61012) in accordance with the manufacturer's instructions. cDNA was synthesized with a High Capacity cDNA Reverse Transcription Kit (ABI, Cat No. 4368814) according to the manufacturer's instructions. Reaction conditions were as follows: 25°C for 10 min, 37°C for 2 h, 85°C for 5 min, and 4°C for ∞. The reverse transcription reaction were performed without pure RNA samples (no reverse transcription control) to determine that the prepared mRNA samples did not comprise genomic DNA. We used Xeno RNA (SYBR Green Cell-to-CT Control Kit, Life Technologies, Cat. No. 4402959) as an external reference in the sample for proportionate 1 µl/20 embryos before mRNA extraction. The expression of Xeno RNA in each sample was detected after reverse transcription to ensure the efficiency of mRNA extraction and cDNA reverse transcription in each sample. The cDNA samples were placed on ice directly for qPCR reactions, and spare samples were stored at −20°C.

### Primer design

We researched the recent literature and selected 12 candidate genes: *β-actin*, *Gapdh*, *H2afz*, *Tbp*, *Hprt*, *Ywhaz*, *Pgk1*, *Ubc*, *Ppia*, *Ppib*, *16s*, and *18s* (Table 1). These genes belong to several functional classes and should not be coregulated, thus providing a reliable method of normalizing qPCR expression data. The primers for the 1 target gene (*Oct4*) and 12 candidate reference genes were designed using the primer analysis software Primer3 (http://frodo.wi.mit.edu/primer3/; Table 2). The gene specificities of the primer sequences were confirmed by BLAST searches, and the primers spanned at least two exons or had a large intron sequence between the sense and antisense primer to avoid false-positive amplification of contaminating genomic DNA in the mRNA samples. Amplification efficiencies (E values) and correlation coefficients (R^2^ values) of the 13 genes were obtained using the slopes of the standard curves (Table 2). Only Cq values less than 35 were used to calculate the R^2^ values and E values.

**Table 1:** Candidate reference genes evaluated in this study.

Symbol	Gene name	Function	Localization
β-actin	Actin Beta	Cytoskeletal structural protein	Chromosome 5
Gapdh	Glyceraldehyde-3-phosphate dehydrogenase	Glycolytic enzyme	Chromosome 6
H2afz	H2A histone family, member Z	DNA binding;protein heterodimerization activity	Chromosome 3
Tbp	TATA box binding protein	RNA polymerase II repressing transcription factor binding	Chromosome 17
Hprt1	Hypoxanthine guanine phosphoribosyl transferase	Purine synthesis in salvage pathway;purine ribonulceoside salvage	Chromosome X
Ywhaz	TYrosine 3-monooxygenase/tryptophan 5-monooxygenase activation protein, zeta polypeptide	Signal transduction by binding to phosphoserine-containing proteins	Chromosome 15
Pgk1	phosphoglycerate kinase 1	A highly conserved transferase involved in glycolysis that catalyzes the formation of ATP.	Chromosome X
Ubc	Ubiquitin C	Protein degradation	Chromosome 5
Ppia	peptidylprolyl isomerase A	peptidyl-prolyl cis-trans isomerase activity	Chromosome 11
Ppib	peptidylprolyl isomerase B	isomerase activity; peptide binding	Chromosome 9
16s	16S ribosomal RNA	structural constituent of ribosome	Chromosome 7
18s	18S ribosomal RNA	Ribosomal eukaryotic small subunit	Chromosome 6

**Table 2:** Primers for the 13 genes and parameters derived from qPCR data analysis.

Gene	GenBank accession number	Forward primer	Reverse primer	Product size (bp)	PCR efficiency	Regression coefficient (r2)
16s rRNA	NM_013647	AGATGATCGAGCCGCGC	GCTACCAGGGCCTTTGAGATGG	163	99.2%	0.999
18s rRNA	BK000964	CGCGGTTCTATTTTGTTGGT	AGTCGGCATCGTTTATGGTC	219	99.1%	0.999
β-actin	NM_007393.3	GCCAACCGTGAAAAGAT	AGAGCATAGCCCTCGTAGAT	173	93.9%	0.998
Ppia	NM_008907	GAGCTCTGAGCACTGGAGAGA	CCACCCTGGCACATGAAT	85	98.2%	0.999
Ppib	NM_011149	ACGAGTCGTCTTTGGACTCTTT	GCCAAATCCTTTCTCTCCTGTA	88	91.0%	1.0000
Gapdh	NM_008084	AACTTTGGCATTGTGGAAGG	ACACATTGGGGGTAGGAACA	223	92.1%	0.999
H2afz	NM_016750.2	CTGAAGTAGTGGGTTTTGATTG	GGGATATGACCTTTATTGAGCT	147	99.0%	0.998
Hprt1	NM_013556.2	CAGCGTCGTGATTAGCG	GCCTCCCATCTCCTTCAT	160	97.3%	0.999
Pgk1,	NM_008828.2	TGAGGGTGGACTTCAACG	GGCTCATAAGGACAACGG	126	100.5%	1.000
Tbp	NM_013684.3	CCCTTGTACCCTTCACCAAT	GCAGTTGTCCGTGGCTCT	224	99.0%	0.982
Ubc	NM_019639.4	CCCAGTGTTACCACCAAG	ATCACACCCAAGAACAAGC	100	97.6%	0.997
Ywhaz	NM_011740.3	GAAGGGTGATCACTACCGTTAC	TGGGGAGTTCAGGATCTC	193	99.4%	0.998
Oct4	NM_013633	CACGAGTGGAAAGCAACTCA	AGATGGTGGTCTGGCTGAAC	246	104.6%	0.995

### Real-time reverse transcription quantitative PCR

PCR was performed on the Bio-Rad CFX96 Real-Time System (Bio-Rad Laboratories). The PCR reaction consisted of 0.5 µl cDNA sample, 10 µl TransStart Top Green qPCR Super Mix (TransGen, Cat. No. AQ131), and 100 nM of the forward and reverse primers in a total volume of 20 µl. qPCR amplification was performed for 40 cycles, and the conditions were: 94°C for 15 s, 60°C for 15 s, and 72°C for 20 s. Then a melting curve was generated by heating the amplicon from 60°C to 95°C.

The amplification specificity of each qPCR assay was confirmed by melting curve analysis to verify that the primers amplified could form only one specific PCR product. The amplification efficiencies were calculated according to the formula: efficiency (%) = (3^(−1/slope)−1^)×100. The amplification efficiencies of all the tested genes ranged from 90% to 105%, with all correlation coefficients >0.98. These results demonstrated that the synthesized primer sequences were accurate and suitable for the experiments (Table 2). The assay included a no-template control (NTC), which was detected and indicated no amplification. All qPCR reactions were carried out biologically and technically in triplicate.

### Analysis of expression stability

The stability of each candidate gene was analyzed using four separate reference gene stability analysis software programs: geNorm, NormFinder, the comparative delta-Ct method, and RefFinder. All four software programs were used according to the manufacturer's instructions. To analyse statistically significant variability in gene expression levels between each developmental stage, the Student's *t*-test was applied. Differences of *P*<0.05 were considered significant. The difference in mRNA expression was analysed using SPSS 19.0.

## Results

### Gene expression profile analysis in embryos of different ploidies

**Figure 2:**
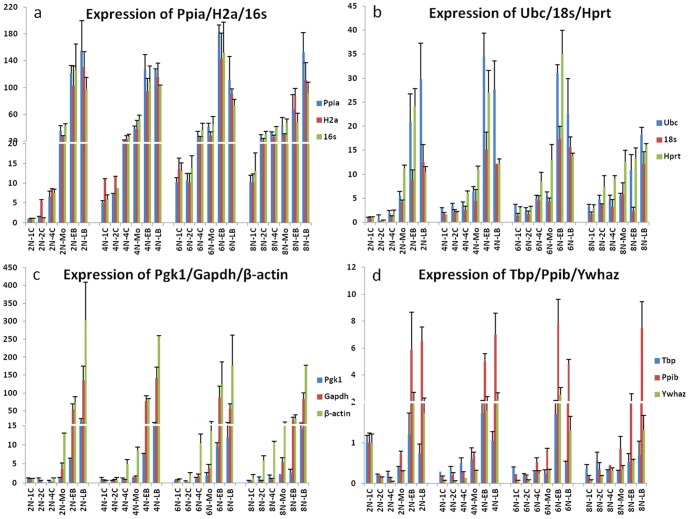
The expression profiles of selected reference genes in embryos of various ploidies by qPCR. The expression patterns of reference genes can be divided into four types. a: The expression levels of *Ppia*, *H2a*, and *16s*. b: The expression levels of *Ubc*, *18s*, and *Hprt*. c: The expression levels of *Pgk1*, *Gapdh* and *β-actin*. d: The expression levels of *Tbp*, *Ppib*, and *Ywhaz*. 2N, diploid; 4N, tetraploid; 6N, hexaploid; 8N, octoploid; 1C, 1-cell; 2C, 2-cell; 4C, 4-cell; MO, morula; EB, early blastocyst; LB, late blastocyst. n = 20.

We compared the expression patterns of reference genes of different ploidies embryos at various developmental stages (four ploidies and six developmental stages). In diploid preimplantation embryos, the transcripts of *Gapdh*, *Pgk1*, *Ywhaz*, *Ppib*, and *Tbp* were decreased from the 1C to 4C stage, and the *Ywhaz*, *Ppib*, and *Tbp* mRNA levels showed a sharp decrease. For the rest of the genes, a minor decrease/increase in the mRNA levels occurred at the 2C stage and was immediately followed by a surge at the 4-cell stage, with a continuous increase thereafter (Figure 2). In the polyploid embryos, most reference genes increased according to the developmental stage, except for *Gapdh*, *Ubc*, *Pgk1*, *Ppib*, and *Tbp*. These patterns varied for a few stages. For example, there was a low expression of *Gapdh*, *Ubc*, *Pgk1*, *Ppib*, and *Tbp* at the 6N-2C stage.

Despite the similar expression patterns were observed between embryos of different ploidies (i.e., a sharp increase from the 4C or Mo stage), from 1C to the LB, the stage-by-stage comparisons revealed differential expression levels. For example, the expression levels of *β-actin* increased sharply by 302-fold; however, the expression levels of *Ywhaz*, *Ppib*, and *Tbp* increased only by 1.7- to 7.9-fold. The expression levels of *18 s*, *Hprt*, *Ubc*, and *Pgk1* increased by 17.4- to 35-fold, and the levels of *H2a*, *16 s*, *Ppia*, and *Gapdh* increased by 141.5- to 180.4-fold.

In summary, the preimplantation development of polyploid embryos was a dynamic process, and time and spatial gene expression patterns were observed. Except for the 1C stage, the expression levels of most genes in the polyploid embryos were higher than that in the diploid embryos. But the rate of increase was disproportionate with the ploidies; the difference in gene expression was not significant between embryos of different ploidies at the morula stage to the blastocyst stage.

### Stability of internal reference genes

In order to identify the most stable reference genes, the 12 reference genes were examined and ranked by the four algorithms (geNorm, NormFinder, the comparative delta-Ct method, and RefFinder) individually. These ranks were summed, with the lowest rank representing the most stable reference gene and vice versa.

#### geNorm analysis

The geNorm program [21], a Visual Basic application (VBA) tool for Microsoft Excel, provides a measure of gene expression stability (M value) based on that the expression ratio of two stable reference genes should be constant in various tissues or under different conditions. The gene with the highest M value is excluded and the new M values of the remaining genes are calculated. The calculation continues until the last two genes are left. The gene with the lowest M value is the most stable, whereas the gene with the highest M value is the least stable.

**Figure 3:**
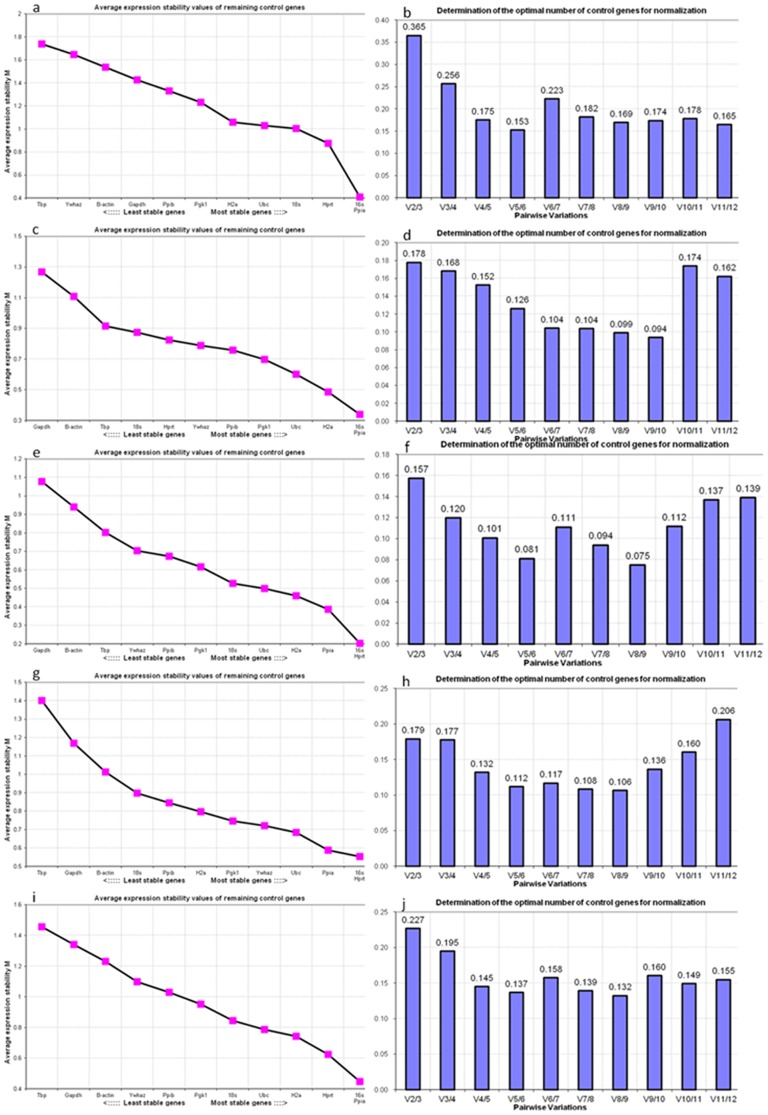
Gene stability values and the optimal number of selected reference genes by geNorm. geNorm analysis of diploid (a, b), tetraploid (c, d), hexaploid (e, f), and octoploid (g, h) embryos and the total sample (i, j). A measure of gene expression stability (M value) of 12 candidate reference genes (a, c, e, g, i). Lower M values indicate more stable expression. Determination of the optimal number of reference genes for normalization was conducted (b, d, f, h, j). The cut-off value was 0.15, which means that if the V value was lower than 0.15, then adding an additional reference gene was not required.

The expression stabilities of the 12 candidate reference genes were analyzed via the geNorm program. The M values of the 12 reference genes from three independent experiments are displayed in Table 3. The reference genes *Ppia*, *16s*, and *H2a* were identified as the three most stably expressed genes in the 4N group (Figure 3c). *Ppia*, *16s*, and *Hprt* were the most stably expressed genes in the 2N, 6N, and 8N groups and in the total sample group (Figure 3a, e, g, and i). The three least stable reference genes in the 2N sample group were *Tbp*, *Ywhaz*, and *β-actin* (Figure 3a). The three least stable reference genes in the 4N, 6N, 8N and the total sample groups were *Tbp*, *Gapdh*, and *β-actin* (Figure 3c, e, g, and i).

**Table 3:** Gene stability value calculated by geNorm.

Rank	2N	M value	4N	M value	6N	M value	8N	M value	Total Sample	M value
1/2	16s/Ppia	0.409	16s/Ppia	0.339	16s/Hprt	0.203	16s/Hprt	0.553	16s/Ppia	0.448
3	Hprt	0.877	H2a	0.486	Ppia	0.388	Ppia	0.587	Hprt	0.625
4	18s	1.003	Ubc	0.601	H2a	0.460	Ubc	0.684	H2a	0.742
5	Ubc	1.029	Pgk1	0.698	Ubc	0.499	Ywhaz	0.721	Ubc	0.786
6	H2a	1.058	Ppib	0.757	18s	0.528	Pgk1	0.747	18s	0.844
7	Pgk1	1.231	Ywhaz	0.788	Pgk1	0.617	H2a	0.798	Pgk1	0.951
8	Ppib	1.330	Hprt	0.825	Ppib	0.674	Ppib	0.845	Ppib	1.028
9	Gapdh	1.428	18s	0.875	Ywhaz	0.704	18s	0.898	Ywhaz	1.098
10	β-actin	1.537	Tbp	0.915	Tbp	0.802	β-actin	1.013	β-actin	1.232
11	Ywhaz	1.647	β-actin	1.108	β-actin	0.941	Gapdh	1.168	Gapdh	1.340
12	Tbp	1.740	Gapdh	1.268	Gapdh	1.077	Tbp	1.402	Tbp	1.457

The optimal number of reference genes required to obtain reliable results from qPCR studies can also be calculated by the geNorm program. The calculation was performed by analysis of the pair-wise variation (V value) of consecutive normalization factors (N_F_) with an increasing number of reference genes (N_Fn_ and N_Fn+1_) (Figure 3). Vandesompele and colleagues proposed using 0.15 as the cut-off value, which means that if the V value is lower than 0.15 then adding an additional reference gene is not required [21]. For example, using only the three top rated reference genes resulted in a value less than 0.15 in the 6N group (V3/4 = 0.12; Figure 3f), therefore, no more reference genes are added to the normalization process in the this group.

#### NormFinder analysis

NormFinder [22] is another VBA program, and it focuses on finding the most stable reference gene and taking into account the intra- and intergroup expression variation. The stability values and standard errors are calculated according to the transcription variation of the reference genes. Stably expressed genes, which have low variation in expression levels, exhibit low stability values.

The NormFinder analysis results of our data were shown in Table 4. The most stable reference genes in the 2N group were *Ubc*, *18 s*, and *Hprt*. *Ppia*, *Ppib*, and *Ywhaz* were the most stable in the 4N group, *Ppia*, *H2a*, and *Ppib* were the most stable in the 6N group, *Pgk1*, *Ppia*, *a*nd *Ywhaz* were the most stable in the 8N group, and *Ubc*, *Pgk1*, and *Ppib* were the most stable reference genes in all samples. The three most unstable reference genes were *Tbp*, *β-actin*, and *Ywhaz* in the 2N group, and *Tbp*, *β-actin*, and *Gapdh* in the 4N, 6N, 8N, and the total sample groups.

**Table 4:** Ranking of the candidate reference genes according to their stability values using NormFinder.

	2N		4N		6N		8N		Total Sample	
Rank	Gene name	Stable value	Gene name	Stable value	Gene name	Stable value	Gene name	Stable value	Gene name	Stable value
1	Ubc	0.256	Ppia	0.213	Ppia	0.145	Pgk1	0.171	Ubc	0.347
2	18 s	0.353	Ppib	0.278	H2a	0.293	Ppia	0.193	Pgk1	0.478
3	Hprt	0.667	Ywhaz	0.370	Ppib	0.299	Ywhaz	0.357	Ppib	0.506
4	Ppib	0.687	Pgk1	0.382	Pgk1	0.339	16 s	0.403	18 s	0.508
5	Pgk1	0.740	Ubc	0.387	Ywhaz	0.344	Ubc	0.449	H2a	0.578
6	H2a	0.841	H2a	0.396	Ubc	0.374	Hprt	0.457	Hprt	0.595
7	16 s	0.874	16 s	0.420	18 s	0.375	Ppib	0.538	16 s	0.625
8	Ppia	0.909	Hprt	0.646	16 s	0.399	18 s	0.585	Ywhaz	0.716
9	Gapdh	0.975	18 s	0.671	Hprt	0.503	H2a	0.665	Ppia	0.771
10	Ywhaz	1.237	Tbp	0.823	Tbp	0.900	β-actin	0.889	β-actin	1.094
11	β-actin	1.322	β-actin	1.219	β-actin	0.942	Gapdh	1.281	Gapdh	1.164
12	Tbp	1.347	Gapdh	1.337	Gapdh	1.145	Tbp	1.706	Tbp	1.284

#### The comparative delta-Ct method

The comparative delta-Ct method [23] is similar to geNorm analysis, by which pairs of genes are compared using delta-Ct approach. This approach is based on the nomenclature and guidelines of the Minimum Information for Publication of Quantitative Real-Time PCR Experiments (MIQE): the quantification cycle (Cq) is preferred to the threshold cycle (Ct), with both describing the fractional PCR cycle used for quantification. Proper investigation of gene expression involves the standardization of the starting mRNA, whereby a variable amount of RNA is added to each reverse transcription reaction. If the ΔCq value between the two genes remains constant, it means that both genes are stably expressed between the samples. However, if ΔCq fluctuates, one or both genes are variably expressed. The addition of a third, fourth, and fifth gene into the comparisons will determine which pairs are low variability, and which genes have stable expression among the samples tested.

The expression level of the 12 candidate reference genes was determined (Figure S1), and the ranking of these genes by the ΔCq approach is shown in Table 5. The three most stable reference genes were *Ubc*, *18 s*, and *Hprt* in the 2N group, *Ppia*, *H2a*, and *16 s* in the 4N group, *Ppia*, *H2a*, and *Ubc* in the 6N group, *Ywhaz*, *Ppia*, and *Pgk1* in the 8N group, and *Ubc*, *Ppia*, and *Pgk1* in the total sample group. The three most unstable reference genes were *β-actin*, *Tbp*, and *Ywhaz* in the 2N group, *β-actin*, *Gapdh*, and *18 s* in the 4N group, and *Tbp*, *β-actin*, and *Gapdh* in the 6N, 8N, and the total sample group.

**Table 5:** Ranking by the average standard deviation using the comparative delta-Ct method.

	2N		4N		6N		8N		Total Sample	
Rank	Gene name	Average of STDEV	Gene name	Average of STDEV	Gene name	Average of STDEV	Gene name	Average of STDEV	Gene name	Average of STDEV
1	Ubc	1.35	Ppia	0.95	Ppia	0.79	Ywhaz	0.99	Ubc	1.14
2	18 s	1.40	H2a	1.01	H2a	0.84	Ppia	0.99	Ppia	1.22
3	Hprt	1.58	16 s	1.04	Ubc	0.90	Pgk1	1.05	Pgk1	1.26
4	Ppib	1.58	Ubc	1.04	Ywhaz	0.91	Ubc	1.10	Ppib	1.27
5	Ppia	1.63	Ppib	1.05	16 s	0.91	Hprt	1.14	H2a	1.27
6	Pgk1	1.64	Ywhaz	1.08	Ppib	0.92	Ppib	1.16	Hprt	1.29
7	16 s	1.67	Pgk1	1.10	Pgk1	0.92	H2a	1.18	16 s	1.33
8	H2a	1.69	Hprt	1.20	Hprt	0.98	16 s	1.24	18 s	1.33
9	Gapdh	1.74	Tbp	1.35	18 s	0.99	18 s	1.43	Ywhaz	1.41
10	Ywhaz	2.00	18 s	1.38	Tbp	1.41	β-actin	1.71	Gapdh	1.76
11	Tbp	2.22	Gapdh	1.86	Gapdh	1.58	Gapdh	1.84	Tbp	1.89
12	β-actin	2.34	β-actin	2.06	β-actin	1.62	Tbp	2.23	β-actin	1.95

#### RefFinder

RefFinder is a web-based comprehensive tool for evaluating and screening reference genes from experimental datasets. It integratesgeNorm, Normfinder, and the comparative delta-Ct method to compare and rank the candidate reference genes. RefFinder assigns an appropriate weight to each reference gene and calculates the geometric mean of the weights to obtain the overall final ranking based on the rankings from each program.

The ranking of the 12 candidate reference genes is shown in Table 6. According to the RefFinder analysis, the most stable three reference genes in the 2N group were *Ubc*, *18s*, and *Hprt*. In the 4N and 6N groups, they were *Ppia*, *16 s*, and *H2a*. In the 8N group, the three most stable reference genes were *Ppia*, *Ywhaz* and *Pgk1*. Lastly, in the total sample group, the three most stable reference genes were *Ubc*, *Ppia*, and *Pgk1*. The three most unstable reference genes were *Tbp*, *β-actin*, and *Ywhaz* in the 2N group, and they were *Tbp*, *β-actin*, and *Gapdh* in the 4N, 6N, 8N, and the total sample group.

**Table 6:** Ranking according to the geomean values using RefFinder.

	2N		4N		6N		8N		Total Sample	
Rank	Gene name	Geomean value	Gene name	Geomean value	Gene name	Geomean value	Gene name	Geomean value	Gene name	Geomean value
1	Ubc	1.71	Ppia	1.00	Ppia	1.44	Ppia	2.29	Ubc	1.71
2	18 s	2.52	16 s	2.76	H2a	2.52	Ywhaz	2.47	Ppia	2.62
3	Hprt	3.00	H2a	3.30	16 s	3.42	Pgk1	2.62	Pgk1	3.48
4	Ppia	3.42	Ppib	3.91	Hprt	4.16	Hprt	3.11	16 s	3.66
5	16 s	3.66	Ubc	4.31	Ubc	4.48	16 s	3.17	Ppib	4.58
6	Ppib	5.04	Ywhaz	5.01	Ppib	5.24	Ubc	4.31	H2a	4.64
7	Pgk1	5.94	Pgk1	5.19	Ywhaz	5.65	Ppib	6.95	Hprt	4.76
8	H2a	6.60	Hprt	8.00	Pgk1	5.81	H2a	7.61	18 s	5.77
9	Gapdh	9.00	18 s	9.32	18 s	7.23	18 s	8.65	Ywhaz	8.65
10	Ywhaz	10.32	Tbp	9.65	Tbp	10.00	β-actin	10.00	β-actin	10.63
11	β-actin	10.97	β-actin	11.32	β-actin	11.32	Gapdh	11.00	Gapdh	10.66
12	Tbp	11.66	Gapdh	11.66	Gapdh	11.66	Tbp	12.00	Tbp	11.66

Finally, we obtained the most stable reference genes based on the rankings from each method. In the 2N group, the most stable reference genes were *Ubc*, *18s*, and *Hprt*, and the least stable genes were *Tbp*, *β-actin*, and *Ywhaz.* In the 4N and 6N groups, the most stable genes were *Ppia*, *16s*, and *H2a*, and the least stable genes were *Gapdh*, *β-actin*, and *Tbp*. In the 8N group, the most stable genes were *Ppia*, *Ywhaz*, and *Pgk1*, and the least stable genes were *Tbp*, *Gapdh*, and *β-actin*. In the total sample group, the most stable genes were *Ubc*, *Ppia*, and *Pgk1*, and the least stable genes were *Tbp*, *Gapdh*, and *β-actin*.

These results show that the most stably expressed reference genes vary among the different methods and ploidies. We hypothesized that the ideal three reference genes could be selected by at least three stability algorithms for individual studies. In our results, *Ubc*, *Ppia*, and *Pgk1* were the three top reference genes in diploid and polyploid embryos. Interestingly, the most frequently used reference genes, *Gapdh* and *β-actin*, were among the least stably expressed reference genes in our study.

### Comparative analysis of gene expression level normalization in diploids

**Figure 4:**
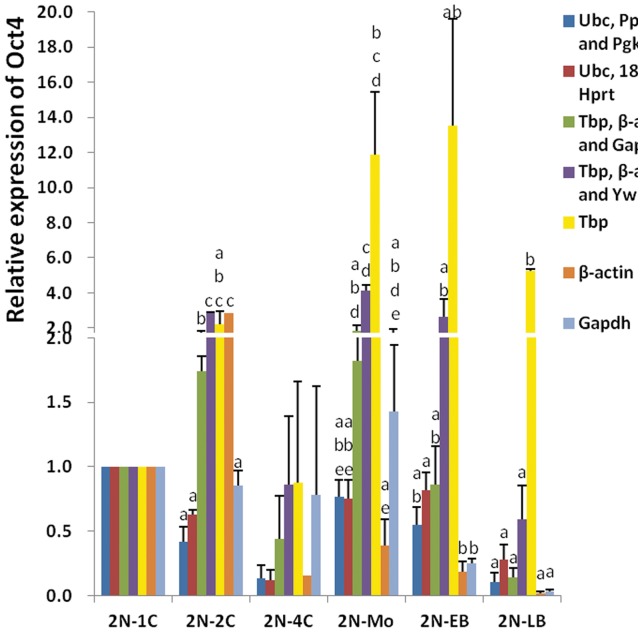
Normalization of *Oct4* gene expression by selected reference genes in diploid preimplantation embryos. *Oct4* expression was normalized to *Ubc*, *Ppia*, and *Pgk1* (dark blue), to *Ubc*, *18 s*, and *Hprt* (red), to *Tbp*, *β-actin*, and *Gapdh* (green), to *Tbp*, *β-actin*, and *Ywhaz* (purple), and to the least three stable reference genes for each developmental stage (*Tbp*, yellow; *β-actin*, orange; *Gapdh*, light blue). 1C, 1-cell; 2C, 2-cell; 4C, 4-cell; Mo, morula; EB, early blastocyst; LB, late blastocyst. Error bars indicate the 95% confidence interval; *P*<0.05 was considered significant; n = 20.

*Oct4* is a major gene associated with the maintenance of pluripotency at each preimplantation stage, and it is expressed as the embryos develop to term. To determine the reliability of different reference gene sets, we compared the relative expression of *Oct4* in diploid preimplantation embryos using different combinations of reference genes. Namely, we compared the relative expression of *Oct4* to the geometric mean of *Ubc*, *Ppia*, and *Pgk1* (the most stable genes in total sample group), the geometric mean of *Ubc*, *18 s*, and *Hprt* (the most stable genes in 2N group), the geometric mean of *Tbp*, *β-actin*, and *Gapdh* (the most unstable genes in total sample group), and the geometric mean of *Tbp*, *β-actin*, and *Ywhaz* (the most unstable genes in 2N group) (Figure 4). Figure 4 exemplifies the variation in the *Oct4* gene expression measured at six developmental stages in the diploid embryos. The levels of the target gene normalized to the geometric mean of *Ubc*, *Ppia*, and *Pgk1* did not differ appreciably from that normalized to the geometric mean of *Ubc*, *18 s*, and *Hprt* at all stages. However, the *Oct4* expression varied significantly when normalization was performed with the *Tbp*, *β-actin*, and *Gapdh* reference gene set, or with the *Tbp*, *β-actin*, and *Ywhaz* reference gene set in several stages. Moreover, there was a significant difference in *Oct4* gene expression in almost every stage when compared with the three least stable genes, except for the 1C and 4C stages. The greatest and lowest *Oct4* expression levels between normalized to the three least stable genes displayed 3.35-, 5.64-, 30.28-, 71.77-, and 276.43-fold in the 2C, 4C, Mo, EB, and LB stages of diploid embryos, respectively. Therefore, our normalization results are reliable.

## Discussion

In this study, we aimed to understand the expression patterns of reference genes in preimplantation embryos of different ploidies at various developmental stages. Therefore, we systematically examined the expression profiles of candidate reference genes in preimplantation embryos of four different ploidies at six different developmental stages. We found that the expression patterns of the reference genes at various embryonic developmental stages roughly divided into four types. Type 1: The expression levels increased dramatically with the progress of embryonic development. Type 2: The expression levels increased moderately at various developmental stages. Although the tendency to increase in type 1 was very similar to that in type 2, the level was different. The extent of the increases in *Ppia*, *H2a*, and *16 s* were more than 100-fold, whereas the increases of *Ubc*, *18 s*, and *Hprt* did not exceed 30-fold. The expression levels of the six genes in the 2N embryos were higher than those in the polyploid embryos until the Mo stage. These genes are relatively more suitable for use as reference genes. Type 3: The expression levels remained constant from the 1C to Mo stages, but increased dramatically at the blastocyst stage. Type 3 included *Gapdh*, *β-actin*, and *Pgk1* for embryos of various ploidies. *Pgk1* expression increased moderately by 20-fold, and *Gapdh* and *β-actin* expression increased up to 100-fold. The sharp increase in gene expression for *Gapdh* and *β-actin* indicated that they are not suitable as reference genes. Type 4: The expression levels were almost immutable although there were slight increases at a few developmental stages. For example, for *Tbp*, *Ppib*, and *Ywhaz*, the expression levels showed no significant increase from the 1C stage to morula stage, and there was a slight increase at the blastocyst stage that was less than 10-fold. These immutable genes are also not suitable for use as reference genes.

Furthermore, after compaction, there was no significant variation in the expression levels between the different ploidies. We speculate that this phenomenon is caused by regulatory mechanisms, such as gene silencing or dosage compensation, that occur after compaction. This result is confirmed by DNA microarray analysis experiments performed at the blastocyst stage [17]–[19].

In the present study, we selected the most stable reference genes from 12 commonly used candidate reference genes in diploid and polyploid mouse preimplantation embryos using geNorm, NormFinder, the comparative delta-Ct method, and RefFinder programs. Although the rankings of the tested reference genes by the four programs showed slightly different patterns, there were similarities in the composition of the highly ranked genes by each program. The differences in the stability rankings of the candidate reference genes may be produced by using the different algorithms and analytical principlesof four programs. Therefore, we selected the most suitable reference genes for the accurate normalization of target gene expression by combining the data obtained for the top three reference genes from each program.

Our results show that the selection of the standardization genes was not identical in embryos of different ploidies using the different programs; however, it was still possible to select common reference genes. In the 2N group, the most stable genes were *Ubc*, *18 s*, and *Hprt*, and the least stable genes were *Tbp*, *β-actin*, and *Ywhaz.* In the 4N and 6N groups, the most stable genes were *Ppia*, *16 s*, and *H2a*, and the least stable genes were *Gapdh*, *β-actin*, and *Tbp*. In the 8N group, the most stable genes were *Ppia*, *Ywhaz*, and *Pgk1*, and the least stable genes were *Tbp*, *Gapdh*, and *β-actin*. In total sample group, the most stable genes were *Ubc*, *Ppia*, and *Pgk1*, and the least stable genes were *Tbp*, *Gapdh*, and *β-actin*. Altogether, *Ubc*, *Ppia*, and *Pgk1* were the most suitable reference genes, and *Tbp*, G*apdh*, and *β-actin* were the least suitable reference genes.

To testify the suitability of the selected reference genes in our study, the expression levels of *Oct4* were measured by normalization with the geometric means of different sets (*Ubc*, *Ppia*, and *Pgk1*; *Ubc*, *18 s*, and *Hprt*; *Tbp*, *β-actin*, and *Gapdh*; and *Tbp*, *β-actin*, and *Ywhaz)*, and with the least three stable genes (*Tbp*, *β-actin*, and *Gapdh*). The expression levels of *Oct4* normalized to the geometric mean of *Ubc*, *Ppia*, and *Pgk1*, or to the geometric mean of *Ubc*, *18 s*, and *Hprt* did not differ appreciably in any of the stages. However, the *Oct4* expression varied significantly when normalized to the *Tbp*, *β-actin*, and *Gapdh* set, or to the *Tbp*, *β-actin*, and *Ywhaz* set at several stages, and the difference in *Oct4* gene expression was significant in almost every stage when compared with the three least stable genes. These results indicated that the reference genes we chose for gene expression quantification in preimplantation development were appropriate. Moreover, latest studies also revealed that normalization of target gene using unstable reference genes led to significantly different results compared with those using suitable reference genes [24]–[26]. Unfortunately, a number of studies still use traditional reference genes, such as*β-actin* and *Gapdh*, or selected a single randomly gene for the normalization of gene expression, and these choices are likely to impair the accuracy of the result [27]–[31]. Therefore, selection of appropriate reference genes is critical to ensure the accuracy of target gene expression quantification using qPCR experiments.

In addition, we chose two pairs of genes in the same class (*16S* and *18S;* and *Ppia* and *Ppib*) of the 12 candidate genes to verify whether coregulated genes affect the fairness of standardized methods. Interestingly, the results showed that when one of coregulated genes was included in the list of the most stable genes, the other one was only ranked in the middle or even close to that of the unstable genes. Therefore, the reference genes will not affect the selection, whether they belong to the same functional categories or not.

## Conclusions

In this study, the expression levels of 12 candidate reference genes were studied in detail in embryos of various ploidies at several developmental stages. The expression patterns of this wide selection of reference genes were compared in diploid and polyploid mouse preimplantation embryos. We chose the three most stably expressed reference genes by using four normalization programs, and we evaluated the stability of the three genes by detecting the *Oct4* expression level during preimplantation development of 2N embryos. We conclude that *Ubc*, *Ppia*, and *Pgk1* are the most stable reference genes for gene expression analysis of mouse diploid and polyploid preimplantation stage embryos.

## Supporting Information

Figure S1 **qPCR Cq values for the 12 reference genes.** Each box plot is based on the biological triplicate mean Cq value for six developmental stages in embryos of various ploidies. Boxes represent the lower and upper quartile ranges, medians are represented by black dashes within boxes, and whiskers indicate the upper and lower data value ranges for the samples tested. 2N, diploid; 4N, tetraploid; 6N, hexaploid; 8N, octoploid.
(TIF)
