# Supplementary figures and images for: Selection and Expression Profiles of Reference Genes in Mouse Preimplantation Embryos of Different Ploidies at Various Developmental Stages
Source: PLoS One. 2014 Jun 13;9(6):e98956. doi: 10.1371/journal.pone.0098956 (PMC4057156; doi:10.1371/journal.pone.0098956)

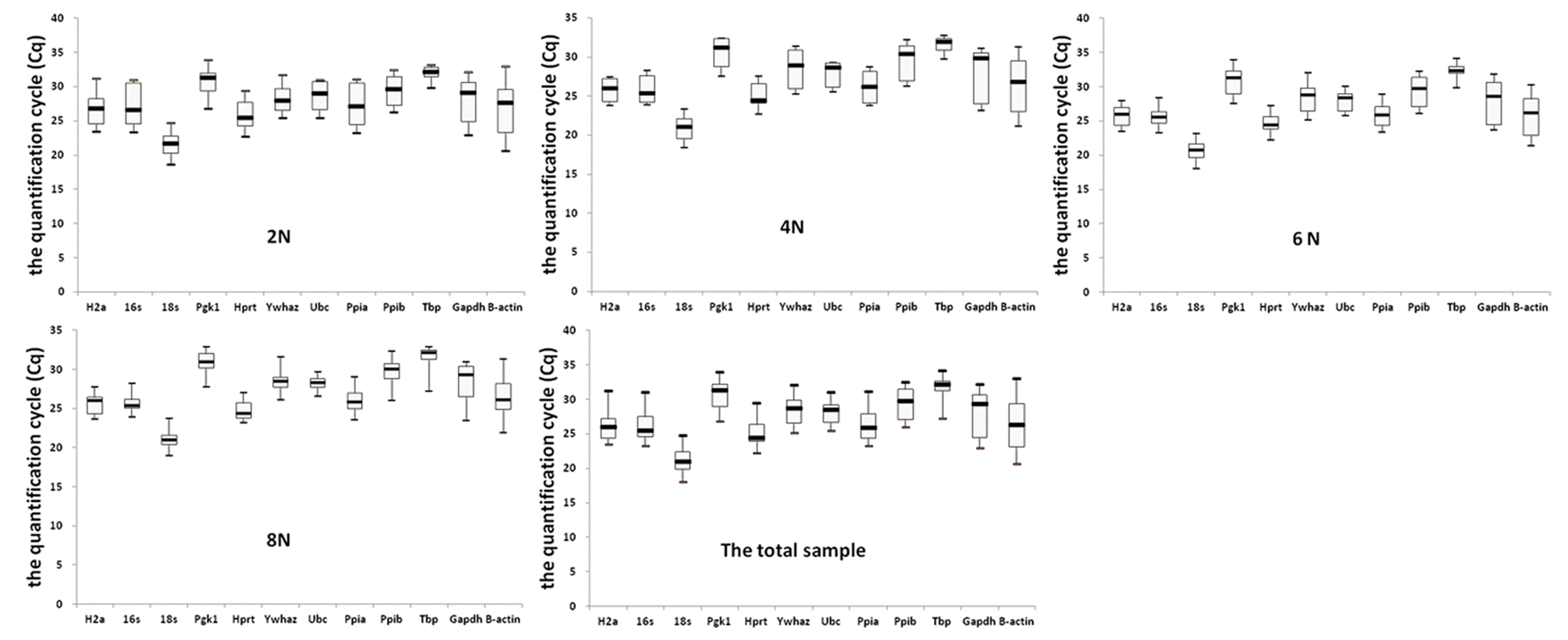

Supplement: Figure S1 — qPCR Cq values for the 12 reference genes. Each box plot is based on the biological triplicate mean Cq value for six developmental stages in embryos of various ploidies. Boxes represent the lower and upper quartile ranges, medians are represented by black dashes within boxes, and whiskers indicate the upper and lower data value ranges for the samples tested. 2N, diploid; 4N, tetraploid; 6N, hexaploid; 8N, octoploid. (TIF) [file pone.0098956.s001.tif]
